# Fecal imaging demonstrates that low‐methoxyl pectin supplementation normalizes gastro‐intestinal transit in mice given a liquid diet

**DOI:** 10.14814/phy2.13662

**Published:** 2018-04-02

**Authors:** Tomohiro Kagawa, Naoyuki Endo, Goro Ebisu, Ippei Yamaoka

**Affiliations:** ^1^ OS‐1 Division Medical Foods Research Institute Otsuka Pharmaceutical Factory, Inc Tokushima Japan

**Keywords:** Enteral nutrition, gastro‐intestinal transit, imaging, methodology

## Abstract

This study has the following aims: (1) to confirm a methodology for a fecal indocyanine green (ICG) imaging test for measuring gastro‐intestinal transit time (GITT); and (2) to compare GITT in mice given a liquid diet in which viscosity increases under acidic conditions to that in mice given stable liquid diets with comparable viscosity or regular chow. To address Aim 1, mice received ICG orally along with intraperitoneal injection of atropine in Study 1, and mice were given ICG orally with concurrent carmine red for Study 2. Fluorescence imaging of feces collected for 8 h thereafter was used to detect the first feces with fluorescence and thereby determine GITT. To address Aim 2, mice were fed ad libitum for 1 week with either liquid diet or regular chow for Study 3, or with liquid diet containing low‐methoxyl (LM) pectin or high‐methoxyl (HM) pectin, or regular chow for Study 4. GITT was then determined by fecal ICG imaging. Atropine delayed GITT in a dose‐dependent manner. The GITT of ICG completely corresponded to that of carmine red (correlation coefficient, 1.00). The first ICG excretion in the loose/some diarrheal feces of mice given a liquid diet was seen at 170 min. Feces of mice given liquid diet were loose with LM pectin and loose/some diarrhea with HM pectin. GITT of mice given liquid diet with HM pectin was significantly delayed (280 min) compared to that of mice given liquid diet with LM pectin (111 min) or regular chow (130 min). Fecal imaging of ICG enables measurements of GITT. LM pectin supplementation in a liquid diet may normalize GITT in mice to that of a normal meal and may be associated with changes in fecal properties.

## Introduction

The onset of bowel movement disorders after starting liquid diets is likely due to the very low fiber content, leading to worsening of the enteral environment (Chang and Huang 2013; Kamarul Zaman et al. 2015). The intake of insoluble fiber appropriately increases the stiffness of the fecal mass (Khoshoo et al. 2010), whereas soluble fiber is fermented by microflora and produces short‐chain fatty acids in the colon, where they act to stimulate fluid and electrolyte absorption (Spapen et al. 2001). Fiber supplementation may thus be beneficial in improving fecal inconsistencies in patients receiving enteral nutrition (EN) formula. Although increased fluidity of fiber‐free liquid diet in the gastro‐intestinal tract may produce the formation of liquid stool and increase stool transit through the gastrointestinal tract, the relationship between gastro‐intestinal transit and stool formation has not been fully examined, to the best of our knowledge.

Pectin is a soluble polysaccharide extracted from citrus fruit such as lemon and lime, and is a linear chain molecule consisting of galacturonic acid and galacturonic methyl‐ester. The degree of esterification of the constituent sugar can generally divide pectin into low‐methoxyl (LM; <50%) or high‐methoxyl (HM; ≥50%) categories, showing differing characteristics of gelation and physical properties of the gel forms. LM pectin forms a gel in the presence of divalent cations, while HM pectin gelates under conditions of high (≥50%) sugar content and low (<3.5) pH (Brejnholt 2010). We developed a liquid EN product containing LM pectin, in which the EN forms a gel under acidic conditions, as seen in the stomach (Yamaoka et al. 2014).

Near‐infrared imaging is a diagnostic imaging method that utilizes fluorescence in the far‐red spectrum (700–900 nm). Indocyanine green (ICG) is a water‐soluble tricarbocyanine dye that fluoresces in the near‐infrared range, and is a diagnostic agent with various uses in clinical settings, such as hepatic and circulatory function tests, cerebral angiography, and identification of sentinel lymph‐nodes. Intravenously injected ICG distributes into the bile at a high rate and thereafter transits the small intestine, cecum and colon, without entering the systemic circulation.

A method to evaluate gastrointestinal transit time (GITT) was devised based on the exclusion of ICG from the systemic circulation and excretion to the feces, with the time from ICG gavage to first appearance of an ICG‐fluorescent fecal pellet indicating the GITT. The aims of the present studies were twofold: first, to confirm a methodology for GITT measurement using ICG fluorescence imaging; and second, to compare GITT between mice given a liquid diet containing no pectin, LM pectin, or HM pectin with that of mice given regular chow.

## Materials and Methods

### Reagent for fecal imaging

ICG (Wako Pure Chemical Industries, Osaka, Japan) was dissolved in distilled water at 0.01% (w/v). This concentration was selected because the greatest amount of fluorescence in EN was observed with 0.01% ICG, compared to 0.001% and 0.1% concentrations (data not shown).

### Animals

Seven‐week‐old BALB/cCr Slc male mice weighing 20–25 g were purchased from Japan SLC (Shizuoka, Japan). Mice were acclimated under ad libitum access to purified solid food (AIN‐93G composition; Oriental yeast, Osaka, Japan) and tap water. Mice were housed in a plastic cage under controlled humidity (55 ± 15%) at room temperature (23 ± 3°C) with a 12:12‐h light‐dark cycle (lights on, 07:00–19:00). The Committee for the Care and Use of Laboratory Animals at Otsuka Pharmaceutical Factory (Tokushima, Japan) approved all experimental procedures used in this study.

### Collection of fecal pellets

**Figure 1:**
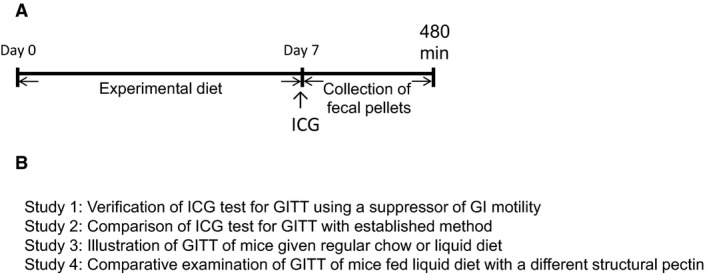
Study overview. (A) Key events associated with measurements by ICG fecal imaging of GITT in mice given experimental diets are shown. On study days 0 through 7, BALB/c mice freely received experimental solid or liquid diet and water, followed by oral ICG administration on day 7. In addition, mice were given intraperitoneal atropine prior to ICG administration in Study 1, and oral carmine red concurrent with ICG in Study 2. Fecal pellets were collected for 8 h after ICG administration and imaging analysis were performed to detect ICG fluorescence. (B) Summary of the aims of Studies 1–4 in this research.

Figure 1 shows the typical experimental design of the present investigation and the research objectives for Studies 1–4.

Study 1 was conducted to address the establishment of the fecal ICG imaging test by confirming that atropine delays GITT. Mice had ad libitum access to food and water and were divided into four groups so that body weight did not differ between groups. Each group of mice received intraperitoneal injections of 110 *μ*L saline (vehicle) or atropine hydrochloride at a dose of 2.5, 5, or 10 mg/kg between 08:30 and 09:00. Mice were orally administered 100 *μ*L of ICG solution 30 min after atropine treatment. Excreted feces were collected from individually housed animals over an 8‐h period following this administration. Feces collection occurred during the lights‐on period under withdrawal of food and water for the 8‐h collection period, and feces were collected on the same conditions in the following studies. Collection time for each fecal pellet was recorded and collected samples were placed into individual wells of a 96‐well plate (MS‐8496F; Sumitomo Bakelite, Tokyo, Japan) and stored on ice with protection from light.

Study 2 compared the fecal ICG imaging test for measuring GITT to the established method using carmine red (Nagakura et al. 1996). ICG and carmine red (Tokyo Chemical Industry, Tokyo, Japan) were dissolved in distilled water at 0.01% (w/v) and 6% (w/v), respectively. Mice (*n* = 11) had ad libitum access to food and water and were orally administered 300 *μ*L of solution containing both ICG and carmine red at around 09:00. As described in Study 1, fecal pellets were subsequently collected for 8 h with recording of collection times under withdrawal of food and water. The interval between gavage and first appearance of a fecal pellet colored with carmine red (red) was determined as the GITT of carmine red. The appearance of carmine red was based on visual observation, and the correlation with the GITT of ICG from the ICG imaging test was assessed.

In Study 3, the GITT of mice given a liquid diet was compared with that of mice given regular chow under the newly established procedure for determining GITT. Each group of mice (*n* = 8 per group) received ad libitum access to water and either solid purified diet (AIN‐93G; Oriental yeast, Osaka, Japan) or pectin‐free liquid diet (Table 1), for 1 week. The composition of the liquid diet and the pectin content were the same as the product (Hine E‐gel; Otsuka Pharmaceutical Factory) used for human enteral feeding. As described in Study 1, 0.1 mL of ICG solution was administered at around 09:00 and fecal pellets were subsequently collected for 8 h under withdrawal of food and water, with recording of collection times and scores for fecal consistency (Score 0, normal; Score 1, loose stool; Score 2, loose/some diarrhea; Score 3, diarrhea) (Fitzpatrick et al. 2012).

**Table 1:** Energy content, macronutrient composition, fiber, and viscosity of enteral nutrition formulae

	EN without pectin	EN with LM pectin	EN with HM pectin
Energy, kcal/100 mL	78.6	80	80
Protein; carbohydrate; fat, g/100 kcal	4.0; 15.9; 2.2	4.0; 16.8; 2.2	4.0; 16.8; 2.2
Total dietary fiber, g/100 kcal	0.5	1.4	1.4
Pectin^a^, g/100 kcal	0	0.9	0.9
Viscosity of EN, mPas·s^b^	4.9	9.0	161.8
Calcium, mg/100 kcal	58.8	58.8	58.8

EN, enteral nutrition; LM pectin, low‐methoxyl pectin; HM, high‐methoxyl pectin.

a Calculated pectin calories.

b Measured at 25°C using a Brookfield viscometer.

In Study 4, the GITT of mice given a liquid diet in which viscosity increased under acidic conditions was measured in comparison with that in mice given a comparably stable liquid diet under such conditions. Each group of mice (*n* = 8 per group) received ad libitum access to water and a liquid diet supplemented with low‐methoxyl (LM) or high‐methoxyl (HM) pectin, or liquid diet completely lacking pectin (Table 1) for 1 week. Oral ICG administration and fecal pellet collection were conducted in the same fashion as in Study 3.

### Fecal imaging and GITT

The distribution of ICG fluorescence in fecal pellets was monitored using the IVIS^®^ Spectrum live animal imaging system (Perkin Elmer, Boston, MA) with excitation and emission at 745 and 840 nm, respectively. Photographic images were analyzed using IVIS^®^ imaging software (Perkin Elmer). The entire area of feces‐containing wells was used as the region of interest (ROI) and the total fluorescent intensity was determined.

The predefined cut‐off value in each mouse based on visual observation was temporarily set by ROI values of all fecal pellets the mouse excreted. Using the predefined cut‐off value, fecal pellets in each mouse were assigned as with (point: 1) or without (point: 0) fluorescence. Receiver operating characteristic (ROC) curves generated from the ROI values and the points (0 or 1) indicate a common cut‐off ROI value in a group to maximize the odds ratio and minimize the risk of errors based on subjective visual evaluation (observer bias). This has the merit of applying individual cut‐off values to that within a group. In detail, after selecting the fecal pellet with the most intense fluorescence among feces collected from the same individuals, feces with ≥20% of maximal intensity were designated as positive for fluorescence. ROC curves were made after measurement of fecal fluorescence and determination of whether feces were positive or negative for fluorescence. As fecal color depended on the diet given to each group, ROC curves were prepared for each group of mice except for Studies 1 and 2. Values for the area under the ROC curve (AUC) for fecal pellets with or without fluorescence in respective groups were of similar range (>0.5) and are shown in Table 2. We set cut‐off values proximate to the coordinate located in the upper‐left part of the ROC curve, and the interval between ICG gavage and appearance of the first fluorescent fecal pellet exceeding the cut‐off was determined as the GITT. Ekuseru‐Toukei 2015 software (Social Survey Research Information, Tokyo, Japan) was used to prepare ROC curves in this study.

**Table 2:** Results of ROC curve analysis for determining cut‐off values in a group for fecal pellets with or without fluorescence

Group		Cut‐off value	Sensitivity/Specificity (%)	AUC	SE (AUC)	*P*‐value	95% CI (AUC)	
Study 1		1.5E + 08	93/99	0.9553	0.0168	<0.001	0.9223	0.9882
Study 2		4.2E + 08	97/97	0.9957	0.0028	<0.001	0.9902	1.0012
Study 3	Regular chow	2.0E + 08	100/100	1.0000	0.0000	<0.001	1.0000	1.0000
Study 3	EN without pectin	4.1E + 08	94/98	0.9850	0.0114	<0.001	0.9626	1.0074
Study 4	Regular chow	2.3E + 08	100/98	0.9986	0.0013	<0.001	0.9960	1.0012
Study 4	EN with LM pectin	3.7E + 08	100/94	0.9912	0.0039	<0.001	0.9836	0.9988
Study 4	EN with HM pectin	4.5E + 08	85/97	0.8765	0.0383	<0.001	0.8015	0.9515

AUC, area under the curve; SE, standard error; CI, confidence interval; EN, enteral nutrition; LM, low‐methoxyl; HM, high‐methoxyl.

### Statistical methods

All values are presented as means ± SD. Differences between groups were analyzed by one‐way analysis of variance (ANOVA) followed by Dunnett's test (Study 1), unpaired Welch's *t*‐test or Student's *t*‐test (Study 3), or Tukey's test (Study 4), as appropriate. Levels of statistical significance were set at: *P < 0.05; and **P < 0.01. Different letters indicates significant differences (*P* < 0.05). Ekuseru‐Toukei 2015 software was used for all statistical analyses.

## Results

**Figure 2:**
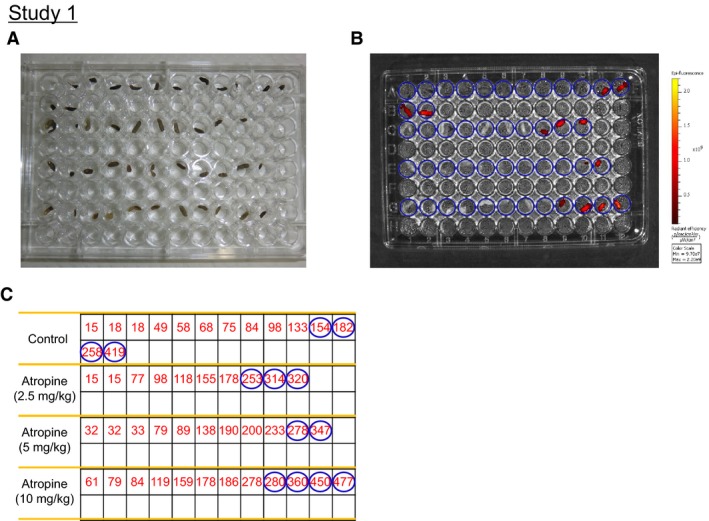
Fecal imaging of indocyanine green to acquire whole gastrointestinal transit time in atropine‐treated (0, 2.5, 5, 10 mg/kg; i.p.) mice in Study 1. (A) Representative visible‐light images of all fecal pellets collected over the experimental period. (B) Fluorescence images of fecal pellets shown in (A) acquired using in vivo imaging at excitation and emission wavelengths of 745 and 840 nm, respectively. (C) Fecal pellets collected into individual wells, starting from the left of each row and continuing into the following row where necessary. The number in the figure shows the time of fecal excretion after indocyanine green gavage (minutes).

Figures 2A and B show visible‐light and fluorescence images, respectively, of fecal pellets excreted from representative ICG‐treated mice, with or without treatment with different doses of atropine. Although excretion of fecal pellets without fluorescence continued for a length of time, fecal pellets yielding fluorescence eventually emerged and their presence continued through the subsequent observation period. The time to first emergence with fluorescence after ICG gavage was defined as the GITT. Table 3 shows the average reading from Figure 2C for six mice. Atropine treatment significantly extended GITT in a dose‐dependent manner, compared to controls without atropine treatment (Table 3).

**Table 3:** Gastro‐intestinal transit time (GITT) in atropine‐treated mice

Study 1	Control	Atropine (2.5 mg/kg)	Atropine (5 mg/kg)	Atropine (10 mg/kg)
GITT, min	130 ± 29	211 ± 27	248 ± 63^a^	327 ± 95^a^

Data are presented as mean ± SD (*n* = 6/group).

a *P* < 0.01 (vs. Control group).

**Figure 3:**
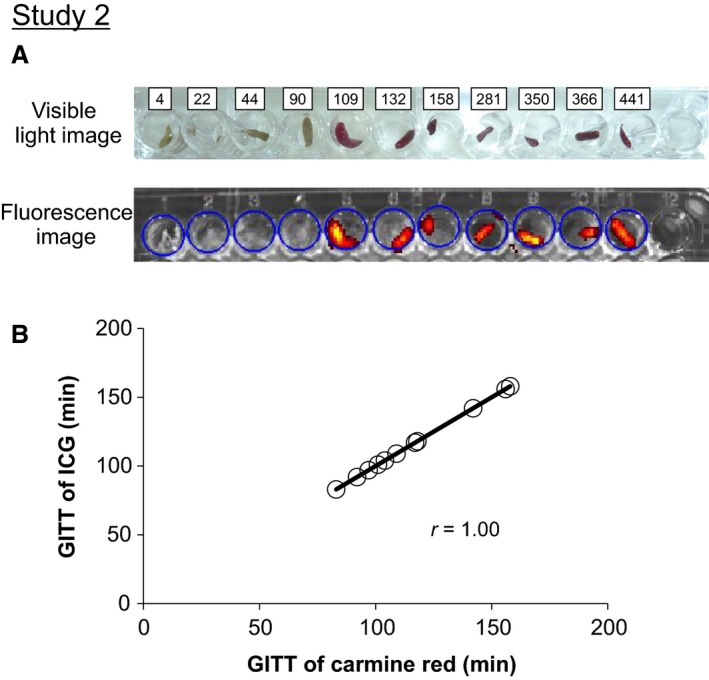
Correlation between GITT as determined using ICG and carmine red. (A) Representative visible‐light images (upper figure) and fluorescence images (lower one) acquired at excitation (745 nm) and emission (840 nm) wavelengths of all fecal pellets collected over the experimental period in Study 2. (B) Correlation analysis of GITT variations in mice (*n* = 11) given oral ICG and carmine red simultaneously. X‐ and Y‐axes represent GITT in mice as measured by carmine red and ICG test, respectively. The *r* in the figure represents the correlation coefficient.

Figure 3A shows representative visible‐light and fluorescence images of fecal pellets excreted from both ICG‐ and carmine red‐treated mice. Excretion of a fecal pellet with simultaneous carmine red and fluorescence was seen, and continued through the subsequent observation period. The GITT of ICG corresponded to that of carmine red (correlation coefficient, *r* = 1.00; Figure 3B).

**Figure 4:**
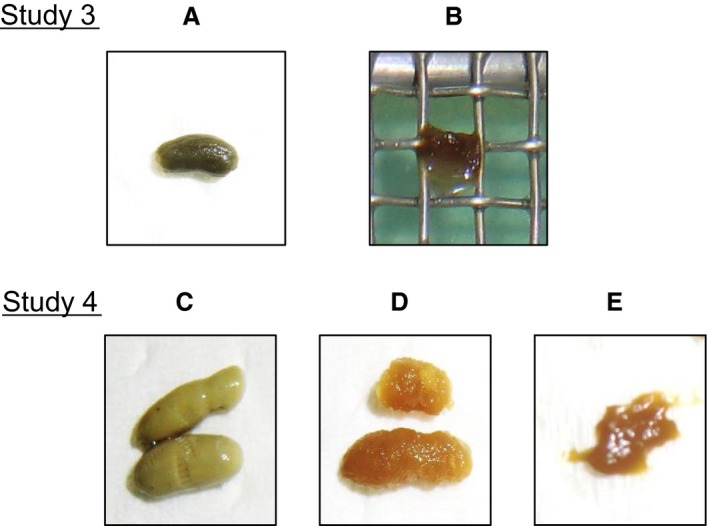
Representative fecal pellets of mice following 1 week of regular chow (A or C), liquid diet lacking pectin (B) or liquid diet containing either low‐methoxyl pectin (D) or high‐methoxyl pectin (E). The shape of individual feces was scored as follows: 0, normal (representative images A and C); 1, loose stool (representative image D); 2, loose/some diarrhea (representative images B and E); 3, diarrhea.

Figure 4 shows a picture of fecal pellets excreted from mice given regular chow, liquid diet containing HM or LM pectin, or liquid diet without pectin. When compared to control feces, which exhibited a solid, elastic form (normal), mice administered the LM pectin‐supplemented liquid diet were softer, but maintained a typical shape (loose stool). In contrast, feces from mice administered pectin‐free or HM pectin‐supplemented liquid diet exhibited a muddy or watery characteristic (loose/some diarrhea). Observed fecal scores in descending order were as follows: HM pectin; LM pectin; and regular chow (Table 4). Fecal scores for mice receiving liquid diet without pectin were in the same range as mice receiving liquid diet with HM pectin (Table 4). GITT was significantly longer in mice given EN without pectin than in controls given regular chow (Table 5). GITT of mice given a liquid diet with LM pectin did not differ from that of controls, but GITT of mice given the HM pectin‐supplemented liquid diet was significantly delayed compared to that of the other two groups (Table 5).

**Table 4:** Fecal scores from Studies 3 and 4

Study 3	Regular chow	EN without pectin
Stool score	0.0 ± 0.2	2.1 ± 0.2^a^
Total number of fecal pellets	107	80

Study 4	Regular chow	EN with LM pectin	EN with HM pectin
Stool score	0.1 ± 0.3^a^	0.9 ± 0.3^b^	1.9 ± 0.4^c^
Total number of fecal pellets	126	209	137

Individual fecal pellet shape was scored as follows: 0, normal; 1, loose; 2, loose/some diarrhea; 3: diarrhea. Data are presented as mean ± SD (*n* = 8/group). Different letters indicate significant differences among groups (*P* < 0.05).

a *P* < 0.01 (vs. regular chow group).

**Table 5:** Gastro‐intestinal transit time (GITT) in mice following 1 week of regular chow or enteral nutrition

Study 3	Regular chow	EN without pectin
GITT, min	153 ± 43	323 ± 53^a^

Study 4	Regular chow	EN with LM pectin	EN with HM pectin
GITT, min	130 ± 36^a^	111 ± 35^a^	280 ± 96^b^

Data are presented as mean ± SD (*n* = 8/group).

a *P* < 0.01 (vs. Regular chow group).

Different letters indicate significant differences among groups (*P* < 0.05).

## Discussion

This study demonstrated that fecal imaging of ICG, a fluorescent probe that does not enter the systemic circulation, enables determination of GITT in mice. Administration of atropine, which suppresses gastrointestinal motility, significantly delayed the first incidence of ICG in fecal pellets of mice fed regular chow in a dose‐dependent manner. Using this fecal ICG imaging system, we evaluated GITT in mice fed a liquid diet supplemented with two different pectins in parallel with scoring for fecal consistency. As a result, mice given an LM pectin‐supplemented liquid diet in which viscosity increased under acidic conditions, such as in the stomach, exhibited similar GITT and fecal pellet shape to controls given regular chow. In contrast, GITT of mice given liquid diet with HM pectin or without pectin, in which viscosity did not appreciably change, was longer than in control and LM pectin groups, and feces became loose or diarrheal.

The botanical extract atropine is a muscarinic receptor antagonist that suppresses gastrointestinal motility in humans following ingestion. The effects of atropine are also known to occur in rodents, where delayed GITT gastrointestinal motility has been reported (De Backer et al. 2008). The findings of the present study confirm the reliability of our ICG method for determining GITT in mice. Specifically, intraperitoneal administration of atropine significantly delayed GITT in mice. Furthermore, a marked delay in GITT was observed when the dosage of atropine was increased. Although GITT has been examined using various methodologies, including methods using Evans blue (Anitha et al. 2016), charcoal suspensions (Touw et al. 2017), and carmine red (Thys et al. 2015), mean GITT in mice measured by ICG imaging in the present study closely corresponded to previously reported values in mice. In fact, this study clearly showed that GITT in mice as measured by ICG imaging corresponded to the GITT obtained using the carmine red method (Fig. 3B). A major advantage of this fluorescence‐based method is the real‐time acquisition of images that are unaffected by fecal pellet color, because this system utilizes a near‐infrared probe that readily eliminates background and autologous fluorescence. The first and second study thus support the conclusion that fecal ICG imaging adequately reflects GITT in mice with or without disturbances in gastrointestinal motility. Measurement of gastrointestinal transit or motility by in vivo ICG fluorescence measurement has been reported elsewhere (Behrendt et al. 2004; Kwon and Sevick‐Muraca 2011). Measurement of bowel length as visualized by ICG indicating relocation within a predetermined interval can evaluate GITT in rats (Behrendt et al. 2004). However, this procedure requires laparotomy and eventration of the intestines, even though intraluminal passage of ICG is easily visualized using an IC‐VIEW system. On the other hand, Kwon and Sevick‐Muraca (2011) noninvasively demonstrated gastric motility (segmental contraction and peristaltic waves) by visualizing movement of ICG fluorescence at a certain ROI, although that methodology has limitations in that animals have to be anesthetized to keep the intestinal lumen in the same location. Our study determined GITT using fecal pellets containing ICG after passing through the entire gastro‐intestinal tract. Therefore, simple collection of fecal pellets should enable measurement without putting the animal in a machine for fluorescence imaging.

We utilized a pectin‐free liquid diet to determine the effect of fiber on GITT and fecal consistency. In fact, most commercially available EN contains relatively small amounts of fiber in the formula because of the effects on viscosity, which reduces flow and causes clogging in feeding tubes. A previous comparative study with liquid meals indicated that solid meals prolong the postprandial period of small intestinal contraction, as well as producing more frequent contractions (Schönfeld 1997). Sustainable postprandial contraction of the small intestine might thus cause smooth ICG excretion in mice given regular chow compared to the pectin‐free liquid diet in the present study. The effect on GITT of adding fiber to liquid diets remains controversial. Meier et al. showed that the addition of soluble fiber (guar gum) did not affect orocecal transit as measured by the hydrogen breath test in human volunteers. A previous study reported that pectin did not affect mouth‐to‐cecal transit time in humans, but the type (LM or HM) of pectin used was not specified (Jenkins et al. 1978). The effect on GITT cannot be measured using the hydrogen breath test without considering the impacts on absorption from the intestine and systemic metabolism. Furthermore, as humans eat food at wide intervals but mice do not, the difference between eating habits is likely to affect GITT. In mice, the stomach seems to be always full during eating, and the colon is much shorter. Different fiber types, different methodologies of determining GITT, and differences between animal species might thus all contribute to differences in results.

We prepared two types of pectin to compare the effects on GITT in mice given EN. As a result, despite supplementing the formula with the same amount of pectin, GITT in mice supplemented with LM pectin was reduced compared to that with HM pectin. Compared to HM pectin, the viscosity of the liquid diet with LM pectin increased when mixed with artificial gastric juice. Furthermore, the gastric contents of mice given liquid diet with LM pectin formed a gel, unlike the gastric contents of mice receiving HM pectin‐supplemented liquid diet (data not shown). Independent of delayed gastric emptying due to increased viscosity by fiber supplementation, LM pectin can increase the frequency and strength of intestinal motility (Xu et al. 2005). Furthermore, another study using rodents showed that when pectin solution was added to homogenized baked beans, movement through the small intestine was faster than in controls without pectin; furthermore, they showed using radioactively labeled meals that food entered the cecum and colon faster. In that study, researchers used citrus‐pectin with a methoxyl content of 7.7%, which would have been considered as LM preparation in our study (Brown et al. 1994). This is further evidence that increased gastric content viscosity by LM pectin might activate intestinal motility and consequently shorten GITT.

This study mainly focused on determining whether the effect on GITT and food consistency occurs with the addition of a specific configuration of pectin. Pectin‐containing liquid diet led to greater basal and butyrate‐stimulated water absorption compared to a fiber‐free control diet in rats (Levine and Rosenthal 1991). Pectin used in that study had a methoxyl content of 6–24% (Levine and Rosenthal 1991). Total short chain fatty acids (SCFAs) in cecal contents were higher in rats given EN with LM pectin than in rats given EN without pectin or HM pectin‐supplemented EN (Yamada et al. 2018). As SCFAs are known to enhance absorption of electrolytes and fluids from the colon, LM pectin might promote fecal bulking caused by colonic fermentation. As stool bulking is known to stimulate colon transit, the similarity in feces shape between mice given liquid diet with LM pectin and those given regular chow may account for the similarity in GITT. This is supported by the finding that reducing GITT by fiber supplementation is often considered related to the effect of fiber on stool bulking (Hillemeier 1995). Furthermore, SCFAs stimulate ileal motility (Kamath et al. 1988) and contribute to reducing GITT in LM pectin‐fed mice. Furthermore, the appearance of a yellow color in the feces of mice given LM pectin (Fig. 4) indicates that bile acid was excreted into the intestine following LM pectin treatment. The ability of pectin to induce bile secretion has been demonstrated in rats (Ide and Horii 1989). Another possible explanation for the reduced GITT is that bile acid produced by LM pectin may contribute to the rapid passage of the liquid diet, because bile acids increase gut motility (Penagini et al. 1988).

In conclusion, fecal imaging employing ICG, a readily available reagent used in multiple clinical applications, permitted simple and accurate recording and analysis of GITT in mice. This method is thus suitable for evaluating drug or diet effects on GITT in mice. Furthermore, the fecal imaging technique was used to demonstrate that changes in fecal consistency exert effects on GITT and, more importantly, that such changes were caused by supplementation with a specific pectin structure in a liquid diet. As gastrointestinal motility is presumably affected by feeding liquid diet, monitoring of motor function in the gut warrants further investigation. These findings may be of some relevance to gastrointestinal dysfunction, particularly dysfunction related to EN feeding, and this mouse model may provide a means for further studies of gastro‐intestinal diseases.

## Conflicts of Interest

None declared except that all authors are employed by Otsuka Pharmaceutical Factory, Inc.
